# Continuous Circulation of Chikungunya Virus during COVID-19 Pandemic in Jambi, Sumatra, Indonesia

**DOI:** 10.3390/tropicalmed7060091

**Published:** 2022-06-05

**Authors:** Marsha Sinditia Santoso, Sotianingsih Haryanto, Fadil Rulian, Rahma F. Hayati, Amanda Kristiani, Rini Kartika, Benediktus Yohan, Martin L. Hibberd, R. Tedjo Sasmono

**Affiliations:** 1 Eijkman Institute for Molecular Biology, National Agency for Research and Innovation, Jakarta 10430, Indonesia; marsha.sinditia.s@gmail.com (M.S.S.); rahma.idn@gmail.com (R.F.H.); benediktus.yohan@gmail.com (B.Y.); 2 Raden Mattaher Hospital, Jambi 36361, Indonesia; sotianingsih@yahoo.com (S.H.); fadilrulianika@gmail.com (F.R.); 3 Dr. Bratanata Hospital, Jambi 36312, Indonesia; amandakristianii@gmail.com; 4 Jambi City Health Office, Jambi 36128, Indonesia; yan.rini@gmail.com; 5 Department of Pathogen Molecular Biology, London School of Hygiene and Tropical Medicine, London WC1E 7HT, UK; martin.hibberd@lshtm.ac.uk

**Keywords:** chikungunya fever, CHIKV, endemicity, Jambi, Indonesia

## Abstract

Chikungunya fever is a self-limiting viral illness that is caused by the chikungunya virus (CHIKV). CHIKV is found in multiple provinces of Indonesia, with clustered local outbreaks. This case series investigates a local chikungunya outbreak during the COVID-19 pandemic, involving two virologically confirmed chikungunya cases found in Jambi, Sumatra, Indonesia in 2021 and the contact tracing of 65 people from the same neighborhood (one of which was also virologically confirmed with CHIKV). The two original cases were symptomatic with classic signs of chikungunya fever, while the CHIKV-positive neighbor was asymptomatic. Out of the 65 participants, chikungunya IgM was detected in seven (10.8%) people while chikungunya IgG was detected in six (9.2%) using capture ELISA. Dengue IgG was detected by rapid test in three (4.6%) of the participants, showcasing a history of dengue virus (DENV) infection along with the circulation of CHIKV in the area. A phylogenetic analysis demonstrates a close evolutionary relationship between all three 2021 Jambi CHIKV isolates and the 2015–2016 isolates from Jambi. This case series showcases the endemicity and persistent circulation of CHIKV in Jambi, leaving the area vulnerable to eminent outbreaks of chikungunya fever and doubling the burden of disease during the COVID-19 pandemic. Health staff training for case detection and notification, as well as an integrated vector surveillance should continue to be implemented to provide an early warning indicator of possible chikungunya outbreaks.

## 1. Introduction

Chikungunya fever is a viral illness caused by the chikungunya virus (CHIKV) of the *Togaviridae* family and *Alphavirus* genus, which is transmitted by the *Aedes* mosquito. It is a self-limiting disease with the most common symptoms being a sudden onset of high fever, severe arthralgia, backache, and headache, while rash is less frequent and is seen in up to 50% of patients [1]. First isolated in Tanzania in 1952, CHIKV is believed to have originated in Africa and has since spread to many regions in Asia and the Pacific [2,3]. CHIKV was first isolated in Indonesia in 1982 in Jambi, Sumatra, and multiple outbreaks have since been reported in other regions of the country [4], as well as the apparent re-emergence of clustered outbreaks since 2001 [5,6]. In 2019, the total number of reported chikungunya cases in Indonesia increased drastically to 5042 cases from 97 cases the previous year [7]. However, in 2020, when the COVID-19 pandemic began to spread in Indonesia and worldwide, the number of reported chikungunya cases in Indonesia dropped to 1689 cases, reported only in the island of Java, while Jambi did not report any cases [7]. This case series investigates the occurrence of a local outbreak of chikungunya in Jambi, Indonesia during the COVID-19 pandemic. In November 2021, Jambi experienced an average of two SARS-CoV-2-positive cases per day, which was relatively low compared to the national average of 402 cases per day [8]. The chikungunya cases that are reported in this study include two virologically confirmed cases found in Jambi as well as the contact tracing of 65 people living in the same neighborhood, one of which was also virologically confirmed to be infected with CHIKV.

## 2. Detailed Case Description

Two cases of suspected chikungunya fever were reported in Raden Mattaher Hospital, Jambi, on 1 November 2021. The first was a 13-year-old male with a 1-day onset of high fever, joint pain, leg muscle pain, nausea, fatigue, and a rash on both hands. A complete blood count showed eosinopenia (0.2%) with other parameters within the normal range. The second case was the first case’s father, a 43-year-old male with a 3-day onset of high fever, severe joint pain, muscle pain, nausea, fatigue, and a rash on the legs. A complete blood count showed leucopenia at 3.96 × 103/uL; neutropenia (43.6%); lymphocytosis (46.8%); and eosinopenia (0.2%), with other parameters within the normal range. Liver function was slightly elevated with ALT (alanine aminotransferase) level at 91 U/L, while kidney function, electrolytes, and glucose levels were normal. Both patients tested negative for CHIKV IgM and IgG using a Standard Q Chikungunya IgM/IgG (SD Biosensor, Suwon, Korea) rapid test; negative for DENV NS1, IgG, and IgM using a Standard Q Dengue Duo (SD Biosensor, Suwon, Korea) rapid test; negative for Zika IgM and IgG using a Standard Q Zika IgM/IgG (SD Biosensor, Suwon, Korea) rapid test; and negative for SARS-CoV-2 IgM and IgG using a VivaDiag COVID-19 IgM/IgG (VivaCheck Biotech, Hangzhou, China) rapid test. Both patients tested positive for CHIKV using a Viasure Zika, Dengue & Chikungunya Real Time PCR Detection Kit (CerTest Biotec, Zaragoza, Spain) with CT values of 27.18 and 25.09 for the first and second cases, respectively. All laboratory tests mentioned above were performed on blood samples that were collected on the day of admission. Convalescent serum of the second case was obtained at 14 days post-fever onset, which tested negative for CHIKV using real-time RT-PCR and positive for CHIKV IgM using a rapid test.

An active case finding was conducted by the Jambi City Heath Office and blood sample collection was performed from people living in the same neighborhood as the two original cases, residing within a 200 m perimeter. Sixty-five serum samples were obtained. Written informed consent was obtained from all participants prior to interview and blood collection. All sera were tested for CHIKV using a Viasure Zika, Dengue & Chikungunya Real Time PCR Detection Kit (CerTest Biotec, Zaragoza, Spain); CHIKV IgG and IgM using a NovaLisa Chikungunya Virus IgG capture and IgM μ-capture ELISA kit (NovaTec Immundiagnostica, Dietzenbach, Germany); and DENV NS1, IgG, and IgM using Standard Q Dengue Duo (SD Biosensor, Suwon, Korea) rapid tests. The summary of the reported symptoms and laboratory results is compiled in Table 1.

The one participant who was positive for CHIKV through real-time RT-PCR also tested positive for chikungunya IgM but did not report any symptoms. Out of the other six participants who tested positive for chikungunya IgM, two reported no symptoms; one reported fever, fatigue, and rash; one reported fever, headache, and myalgia; one reported fatigue, myalgia, arthralgia, and rash; and one reported only myalgia and arthralgia. The presence of dengue IgG in three (4.6%) participants shows that past infection of dengue virus (DENV) is likely. DENV often co-circulates with CHIKV as they share mosquito vector species, i.e., the *Aedes aegypti* and *Ae. albopictus*.

**Figure 1:**
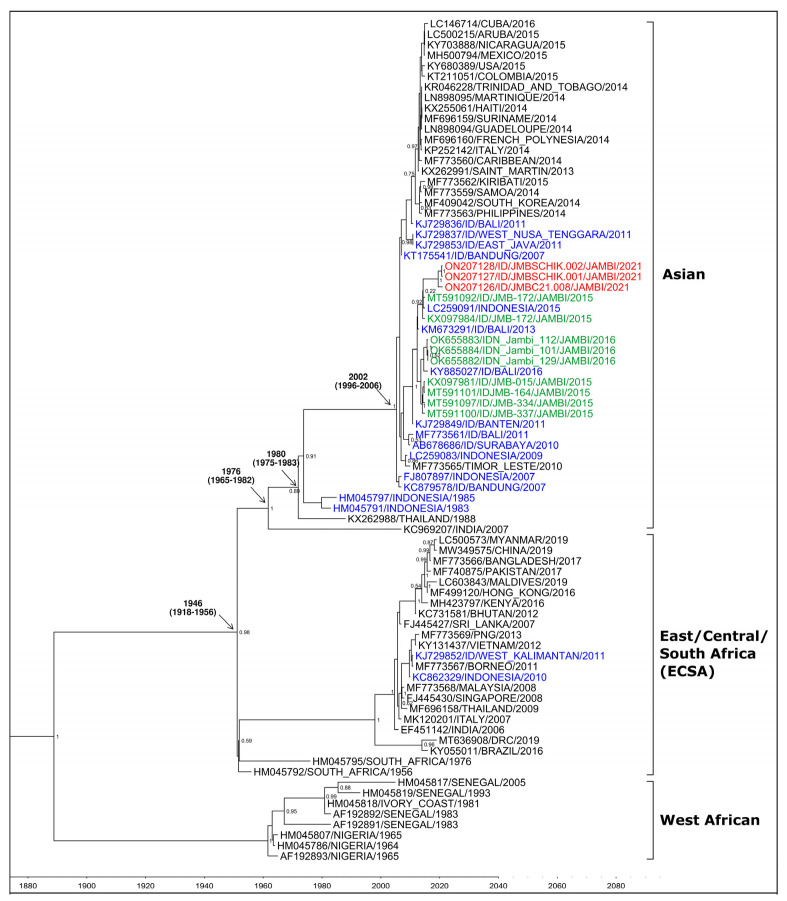
Phylogenetic analysis of CHIKV isolates from Jambi 2021 (red); Jambi 2015 and 2016 (green); other cities in Indonesia (blue); and other countries (black), grouped into genotypes.

Sequencing was then performed on the three RT-PCR-positive samples for CHIKV, targeting the E1 gene using a protocol described elsewhere [3]. Reverse transcription was performed on extracted RNA using Superscript III Reverse Transcriptase (Invitrogen-Thermo Scientific, Waltham, MA, USA). Amplification of the sequences from nt 9870–11,359 was performed using Pfu Turbo DNA Polymerase (Agilent Technologies, Santa Clara, CA, USA) with primer pairs ChikE1/9870F-ChikE1/10710R and ChikE1/10643F-Chik/11359R [3]. Cycle sequencing was performed on these amplified genes using BigDye Dideoxy Terminator kits v.3.1 (Applied Biosystems-Thermo Scientific Waltham, MA, USA). The resulting sequence reads were assembled using the SeqScape v.2.5 software (Thermo-Fisher Scientific, Waltham, MA, USA). The generated consensus sequences of the partial E1 genes of the three CHIKV isolates from Jambi (GenBank accession numbers ON207126, ON207127, ON207128) were grouped with reference sequences that were downloaded from GenBank. A total dataset of 80 sequences was aligned using MEGA X software (Pennsylvania State University, State College, PA, USA) [9] and then analyzed for phylogenetic tree inference using the Bayesian Markov Chain Monte Carlo (MCMC) algorithm with a BEAST v.2.6.6 (University of Auckland, Auckland, New Zealand) [10]. One hundred-million chains were run using a Tamura-Nei model with four gamma parameters (TN93 + Γ4), a relaxed uncorrelated lognormal molecular clock using an initial estimated evolutionary rate of 4.33 × 10^−4^ substitutions [11], and a tree prior set as a coalescent Bayesian skyline plot. The selection of a statistical model for likelihood, optimized for a maximum likelihood (ML) tree, was calculated using MEGA X software (Pennsylvania State University, State College, PA, USA) [9]. To ensure an adequately effective sampling size (ESS) for all parameters after 10% burn-in, the MCMC trace was analyzed using Tracer v.1.7.2 (Andrew Rambaut Group, Edinburgh, UK). A maximum clade credibility (MCC) tree was created and visualized using TreeAnnotator v.2.6.6. (University of Auckland, Auckland, New Zealand) and FigTree v.1.4.4 (Andrew Rambaut Group, Edinburgh, UK). All three Jambi CHIKV isolates belonged to the Asian genotype (Figure 1), with sequence identity and similarity ranging from 98.2% to 99.6% and a normalized global amino acid similarity score ranging from 0.97–0.99 against the strains that were isolated in Jambi in 2015 and 2016 [12].

## 3. Discussion

The three virologically confirmed CHIKV-infected cases and the seven people in the same neighborhood who had antibodies for CHIKV showcase the presence of persistent CHIKV circulation in Jambi, despite the apparent lack of reported cases such as that which occurred in 2020. Phylogeny data show the endemicity of CHIKV in Jambi, with a close evolutionary relationship between the CHIKV that was isolated in 2015–2016 and in 2021 in the city (Figure 1). The classification of Jambi CHIKV isolates into the Asian genotype is consistent with previously reported data on CHIKV phylogeny by our group [13] and others [14], showing that this genotype is still predominantly circulating in the region.

The samples in this study were collected while the COVID-19 pandemic was still ongoing in the country. Studies that were conducted in countries endemic to Chikungunya show that CHIKV continued to circulate and cause disease during the COVID-19 pandemic [15] with possible coinfections [16]. Despite the relatively low incidence of COVID-19 in Jambi during the month in which this study was conducted, the mobility restrictions as a measure of COVID-19 transmission control that are still implemented in cities, including Jambi, create difficulties in other disease surveillance during a pandemic. The mobility restriction also affects the health-seeking behavior of people, which may cause disease underreporting, including chikungunya. Altogether, this creates a double burden in the sense that endemic diseases such as chikungunya fever continue to circulate and affect the community even as the pandemic endures.

## 4. Conclusions

The continuous transmission and circulation of CHIKV in Jambi leave the area vulnerable to eminent outbreaks of chikungunya fever, even during the COVID-19 pandemic. Health staff training to detect and notify clustering cases, as well as integrated vector surveillance should continue to be implemented to provide an early warning indicator of possible chikungunya outbreaks.

## Figures and Tables

**Table 1:** Characteristics of samples used in the study.

Categories	Cases, N = 65 n (%)
*Symptoms*	
History of Chikungunya	0 (0.0)
History of travel out of the city	1 (1.5)
Fever	3 (4.6)
Headache	7 (10.8)
Myalgia	6 (9.2)
Arthralgia	9 (13.9)
Joint swelling	2 (3.1)
Fatigue	4 (6.2)
Rash	2 (3.1)
*Chikungunya Laboratory Results*	
RT-PCR-positive	1 (1.5) *
ELISA IgG-positive	6 (9.2)
ELISA IgM-positive	7 (10.8)
*Dengue Laboratory Results*	
RDT NS1-positive	0 (0.0)
RDT IgG-positive	3 (4.6)
RDT IgM-positive	0 (0.0)

* Ct value 39.3.

## Data Availability

CHIKV sequences were deposited in the GenBank repository with accession numbers ON207126, ON207127, ON207128.
